# Introducing the *f*_0_% method: a reliable and accurate approach for qPCR analysis

**DOI:** 10.1186/s12859-024-05630-y

**Published:** 2024-01-11

**Authors:** Mahmoud Gamal, Marwa A. Ibrahim

**Affiliations:** Department of Biochemistry and Molecular Biology, Faculty of Veterinary Medicine, Cairo University, Giza, 12211 Egypt

**Keywords:** Real-time PCR, Delta C_T_, PCR efficiency, Calibration curve, Performance indicators, Variation between replicates, Curve analysis, Inflection cycle

## Abstract

**Background:**

qPCR is a widely used technique in scientific research as a basic tool in gene expression analysis. Classically, the quantitative endpoint of qPCR is the threshold cycle (C_T_) that ignores differences in amplification efficiency among many other drawbacks. While other methods have been developed to analyze qPCR results, none has statistically proven to perform better than the C_T_ method. Therefore, we aimed to develop a new qPCR analysis method that overcomes the limitations of the C_T_ method. Our *f*_0_% [eff naught percent] method depends on a modified flexible sigmoid function to fit the amplification curve with a linear part to subtract the background noise. Then, the initial fluorescence is estimated and reported as a percentage of the predicted maximum fluorescence (*f*_0_%).

**Results:**

The performance of the new *f*_0_% method was compared against the C_T_ method along with another two outstanding methods—LinRegPCR and Cy_0_. The comparison regarded absolute and relative quantifications and used 20 dilution curves obtained from 7 different datasets that utilize different DNA-binding dyes. In the case of absolute quantification, *f*_0_% reduced CV%, variance, and absolute relative error by 1.66, 2.78, and 1.8 folds relative to C_T_; and by 1.65, 2.61, and 1.71 folds relative to LinRegPCR, respectively. While, regarding relative quantification, *f*_0_% reduced CV% by 1.76, 1.55, and 1.25 folds and variance by 3.13, 2.31, and 1.57 folds regarding C_T_, LinRegPCR, and Cy_0_, respectively. Finally, *f*_0_% reduced the absolute relative error caused by LinRegPCR by 1.83 folds.

**Conclusions:**

We recommend using the *f*_0_% method to analyze and report qPCR results based on its reported advantages. Finally, to simplify the usage of the *f*_0_% method, it was implemented in a macro-enabled Excel file with a user manual located on https://github.com/Mahmoud0Gamal/F0-perc/releases.

**Supplementary Information:**

The online version contains supplementary material available at 10.1186/s12859-024-05630-y.

## Background

Quantitative polymerase chain reaction (qPCR) allows the quantification of minute amounts of a specific target DNA by monitoring the increase in fluorescence associated with its amplification. Fluorescence signals collected in qPCR are commonly produced from DNA-binding dyes, such as SYBR Green I or fluorophore-labeled oligonucleotides [1]. Combining qPCR with reverse transcription (RT-qPCR) extended its ability to quantify RNA, especially microRNA and mRNA. This advent unleashed the power of gene expression analysis which became one of the widely used methods in scientific research [2].

qPCR experiments are used to quantify the target nucleic acid either absolutely or relatively [3]. Absolute quantification requires building a standard curve to give a copy number for each reaction. A standard curve is a linear relationship between the threshold cycles (C_T_) and their *log*_10_ transformed concentrations [4]. On the other side, relative quantification requires one or more reference genes to quantify the target gene relative to them. While absolute quantification is widely used in quantifying microbial nucleic acid, relative quantification is a basic tool in gene expression analysis [3].

The quantitative endpoint of both types of quantification is a threshold cycle (C_T_) that is defined as the PCR cycle at which the fluorescence signal crosses an arbitrary threshold [5]. Till now, C_T_ is the most used method to analyze and report qPCR results [6]. However, there are several limitations to this method including:

1. The efficiency of the PCR reaction (*E*): The PCR reaction starts—in best cases—with (*E* = 2, complete doubling). Then, efficiency gradually declines due to the reduced availability of the reaction substrates till (*E* = 1, no amplification) at the plateau phase [7]. The starting efficiency of the PCR reaction can vary depending on the template, primers, and reaction conditions. However, the C_T_ method assumes that the PCR efficiency is the same for both the target and the reference genes. If there is a significant difference in efficiency between them, then the normalization of the target gene expression using the C_T_ method is invalid [8, 9].
2. Inhibition of the PCR reaction: The presence of inhibitors in the sample can affect the efficiency of the PCR reaction and lead to inaccurate C_T_ values. Inhibitors can be present in the sample due to various reasons such as impurities in the RNA preparation or the presence of PCR inhibitors in the sample matrix [10].
3. Accuracy of the instrument: The accuracy of the qPCR instrument can affect the precision and accuracy of the C_T_ values. Differences in the sensitivity and specificity of the instrument can lead to variations in the C_T_ values obtained [10].
4. Data analysis: The C_T_ method assumes that the PCR amplification is in the exponential phase, and the C_T_ value is determined at a fixed threshold level. However, the actual amplification kinetics can vary between samples and genes, and the choice of threshold level can affect the accuracy of the C_T_ values obtained [11].

Many methods have been developed to overcome the limitations of the C_T_ method such as the sigmoidal models, Cy_0_, LinRegPCR, CyC*, CqMAN, etc. The sigmoidal curve methods involve fitting the raw data to a four or five-parameter sigmoid equation and the initial fluorescence is calculated [12, 13]. In the Cy_0_ method, the raw data is fitted to Richard's equation and a tangent is drawn at the inflection point where its intersection with the abscissa is considered the Cy_0_ value that is used as a C_T_ [14]. LinRegPCR calculates the efficiency for each reaction through a straight line fitted through a predetermined window of linearity. Then, the average of these efficiencies is calculated and used for each amplicon [15]. CyC* determines the earliest amplification cycle (C*) as an outlier over the background fluorescence and calculates efficiency through three amplification cycles starting with the C* followed by calculating the initial template amount [16]. In CqMAN, the Cq is the cycle corresponding to the midpoint between the baseline and the second derivative maximum fluorescence based on a modified Gompertz model. While efficiency (averaged per amplicon) is calculated from a three-parameter exponential model fitted to the cycles from the Cq to the second derivative maximum [17].

Despite the aforementioned limitations of the C_T_ method and the development of many methods to overcome them, the C_T_ method is still the most adopted in analyzing qPCR results [6]. One of the reasons is the simplicity of the C_T_ method. Another important reason is the lack of statistical evidence of the advantage of using the other methods. The C_T_ method was compared once with 10 methods and another time with 13 methods and the Friedman test included the C_T_ method along with LinRegPCR and Cy_0_ in the subset with the highest rank in both studies [7, 16].

The current study aims to develop a qPCR analysis method (*f*_0_%) that addresses the drawbacks of the C_T_ method by minimizing the quantification errors and variation between replicates. Hence, enhances the validity and robustness of the gene expression analysis. Moreover, the performance of the *f*_0_% method was compared with the best methods in analyzing qPCR—the C_T_, LinRegPCR, and Cy_0_ methods—as reported earlier [7, 16] using datasets that depend on DNA-binding dyes. Moreover, the analysis process considered the presence or absence of a dilution curve. Finally, to facilitate the use of the *f*_0_% method, a model was developed and implemented in a user-friendly program.

## Methods

### Datasets

20 dilution curves obtained from 7 different datasets were used to evaluate the *f*_0_% method against the C_T_, LinRegPCR, and Cy_0_ methods. The datasets represent various PCR instruments, DNA binding-dyes, and reaction mixtures. All datasets were imported directly from the qpcR R package [18]. In each reaction, the baseline cycles (C_3:8_) were averaged and their slope was calculated, after which the background fluorescence was subtracted except for the LinRegPCR method. Furthermore, the normalization was performed by dividing the fluorescence of each reaction by the maximum fluorescence of the corresponding dilution curve.

The used datasets are named after the name of their first author as follows:

1. *Boggy* contains a dilution curve with six tenfold dilutions with 2 replicates. The qPCR was performed on Chromo4 thermal cycler (Bio-Rad) using the SYTO-13 fluorescent dye. The target was a randomly generated synthetic DNA sequence that was optimized to reduce the secondary structures [19].
2. *Ruijter* contains a dilution curve with four tenfold dilutions with 94 replicates. The qPCR was performed on CFX 384 instrument (Bio-Rad) using SYBR Green I dye. The DNA target was a synthetic oligonucleotide for the human MYCN gene [7].
3. *Guescini* contains a dilution curve with seven tenfold dilutions with 12 replicates. The qPCR amplification was conducted using LightCycler® 480 (Roche) with SYBR Green I dye. A plasmid containing a 104 bp fragment of the mitochondrial gene NADH dehydrogenase 1 served as the target DNA [14].
4. *Lievens* contains a dilution curve with five fivefold dilutions with 18 replicates. Soybean genomic DNA was used with primers targeting the lectin endogene Le1. Quantification was based on SYBR Green I [20].
5. *Spiess* contains four dilution curves denoted as Spiess_1, Spiess_2a, Spiess_2b, and Spiess_3. Spiess_1 is a dilution curve of seven tenfold dilutions with 4 replicates. Spiess_2a and Spiess_2b are two dilution curves of five fourfold dilutions with 3 replicates for two different cDNA samples. Spiess_3 is a dilution curve of seven fourfold dilutions with 3 replicates. The S27a housekeeping gene served as the target. The qPCR instruments used were LightCycler 1.0 (Roche) for Spiess_1 and MXPro3000P (Stratagene) for Spiess_2a, Spiess_2b, and Spiess_3. While SYBR Green I dye was used for the quantification of all dilution curves, only Spiess_3 was ROX-normalized [18].
6. *Rutledge* contains Six tenfold dilutions with 4 replicates in 5 individual batches. Each batch is considered a dilution curve and denoted as Rutledge_1:5. The primers were designed to amplify a 102 bp amplicon with the help of SYBR Green I dye using Opticon2 (MJ Research Inc) [12].
7. *Vermeulen* is a huge dataset containing the expression data of 59 genes in addition to 5 housekeeping genes. It was performed to build a multigene-expression signature to help in the prognosis of patients with neuroblastoma. Each of the 64 genes had a dilution curve of five tenfold dilutions with 3 replicates. We included only the first 7 genes (alphabetical order) to avoid bias to a single qPCR instrument. The used genes were AHCY, AKR1C1, ARHGEF7, BIRC5, CAMTA1, CAMTA2, and CD44 while ALUsq was excluded as it shows early amplification (C_T_ = 21:26) in the no template control. Quantification was conducted on LightCycler® 480 (Roche) using SYBR Green I dye [21].

### The C_T_ method

The threshold cycle (C_T_) is a method to report qPCR results quantitatively as defined earlier. This method is based on placing an arbitrary threshold at the exponential phase of the amplification curve [5]. Since choosing the threshold value has a great impact on the C_T_ analysis results [11], we employed a strategy that links the threshold to the baseline noise of the corresponding dilution curve. We found that the maximum standard deviation of the baseline fluorescence of each dilution curve serves as a reliable indicator of its noise. Then, the threshold value for each dilution curve was set to be 100-fold the baseline noise. This threshold yielded the best performance in all datasets except for *Boggy*, *Ruijter*, and *Rutledge* datasets where a threshold value of 10, 50, and 50 folds of their baseline noise yielded better results, respectively. An amplification plot for each dilution curve with its threshold is provided in Additional file 1: Fig. S1 and Additional file 2: Fig. S2. Finally, the C_T_ was calculated according to Eq. (1, 2) [22].

1 $$C_{T} = x + \frac{{\ln Threshold - \ln f_{x} }}{\ln E}$$

where *x* is the cycle immediately before the threshold, *f*_*x*_ is the fluorescence at cycle *x*, and *E* is calculated as follows:

2 $$E = \frac{{f_{{\left( {x + 1} \right)}} }}{{f_{x} }}$$

### The LinRegPCR method

The LinRegPCR method analyzes qPCR using efficiencies calculated from the slope of a regression line of the datapoints in the exponential phase of baseline corrected fluorescence data. The software of this method takes non-baseline corrected raw data and corrects the baseline. Then an iterative algorithm is used to allocate the exponential phase known as the window of linearity. Then, the average of these efficiencies is calculated and used for each amplicon. Finally, the software produces an N_0_—initial nucleic acid amount—calculated using the mean efficiency [15].

### The Cy_0_ method

In the Cy_0_ method, the 5-parameter Richard's equation was used to fit a non-linear curve using the raw data. At the inflection point of this curve a tangent is drawn where its intersection with the abscissa is considered the Cy_0_ value [14]. The Cy_0_ was calculated by the cy0() function of the qpcR R package [18] using RStudio-2023.06.1-524 [23] and R programming language v4.3.1 [24].

### The ***f***_0_% method

**Fig. 1:**
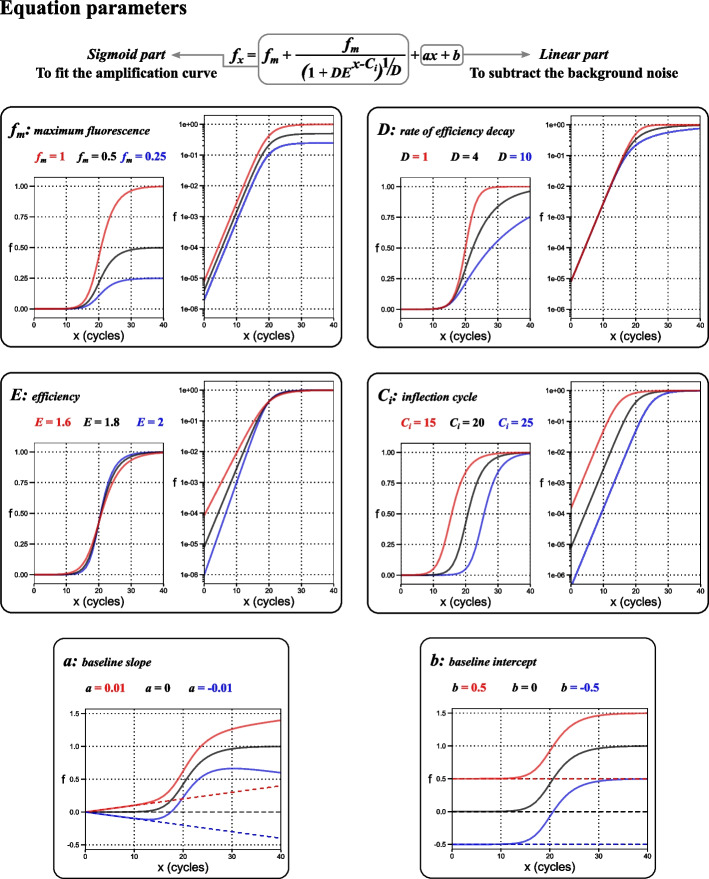
Graphical illustration of the equation parameters. The parameters of the equation could be divided into sigmoidal-related parameters (*f*_*m*_, *D*, *E*, and *C*_*i*_) to fit the amplification curve and linear parameters (*a* and *b*) to fit the baseline of the curve. Where *f*_*x*_ is the fluorescence of cycle *x* and *x* is the cycle number. To illustrate the effect of each of these parameters, the value of only one parameter was varied while maintaining all other parameters to a fixed value (*f*_*m*_ = 1, *D* = 2, *E* = 1.8, *C*_*i*_ = 20, *a* = 0, *b* = 0). All parameters have two plots: linear (left) and logarithmic (right) except for *a* and *b* have only linear plots. *f*_*m*_: the maximum predicted fluorescence. *D*: the rate of efficiency decay, a high value indicates a rapid reduction in efficiency from cycle to cycle. *E*: the starting efficiency or efficiency in the baseline (note that fixing the value of other parameters—specifically *C*_*i*_—while plotting curves with variable *E* necessitates starting with different fluorescence. Thus, low *E* values were compensated with high starting fluorescence leading to an early rise of their amplification curve). *C*_*i*_: the inflection cycle is the point that separates the upper and lower parts of the curve and corresponds to the point with the maximum slope. *a*: baseline slope is the slope of the background noise in case of drifting background. *b*: the baseline intercept is the starting value of the background noise

The *f*_0_% method is based on a six-parameter model Eq. (3) composed of two parts: a four-parameter sigmoid part and a two-parameter linear part. The four-parameter sigmoid part is used to fit the amplification curve with parameters to predict the values of the maximum fluorescence (*f*_*m*_), the rate of efficiency decay (*D*), the starting efficiency (*E*), and the inflection cycle (*C*_*i*_). While the role of the two-parameter linear part is to subtract the background noise with parameters to predict the values of the baseline slope (*a*) and the baseline intercept (*b*). The mathematical role of each of the equation parameters is graphically illustrated in Fig. 1.

3 $$f_{x} = f_{m} - \frac{{f_{m} }}{{\left( {1 + DE^{{x - C_{i} }} } \right)^{1/D} }} + ax + b$$

where﻿ *f*_*x*_ represents the fluorescence at cycle *x*.

The previous equation (Eq. 3) operates in two modes:

1. Free *E* mode: where *E* is a variable that is left to be predicted. Then, *E*s from different reactions are averaged per amplicon to give a prediction of the starting efficiency of that amplicon. This mode of Eq. 3 is fitted to cycles ranging from the first cycle to the cycle just after the inflection cycle (*C*_*i*_).
2. Fixed *E* mode: where *E* is a constant that may be calculated in the free *E* mode or predetermined using the slope of a standard curve as described later. In this mode, all cycles are fitted by solving for *f*_*m*_, *D*, *C*_*i*_,* a*, and *b* using the constant *E*. Then, the initial fluorescence (*f*_*0*_) is calculated using Eq. (4).
  4 $$f_{0} = f_{m} - \frac{{f_{m} }}{{\left( {1 + DE^{{ - C_{i} }} } \right)^{1/D} }}$$

**Fig. 2:**
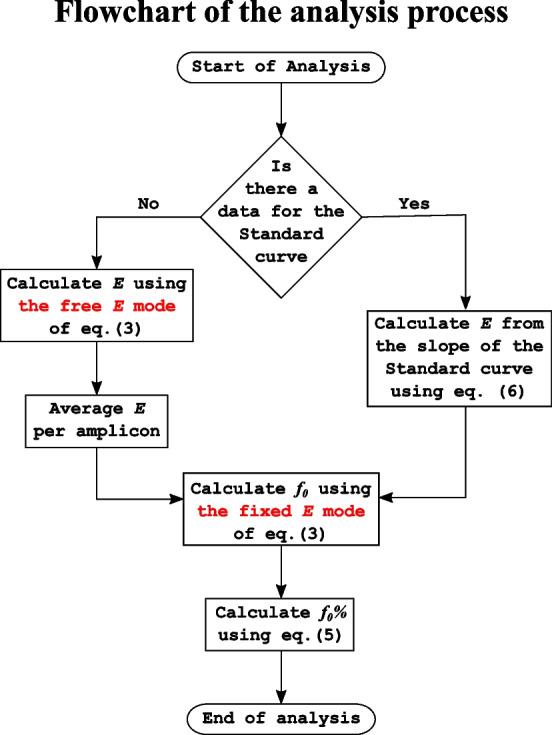
Flow chart of the analysis process. *The first step* is to calculate the starting efficiency (*E*). If there is data for a standard curve, approximate *f*_0_% is calculated using the fixed *E* mode of Eq. (3) assuming (*E* = 2). Then, *log*_10_(*f*_0_%*)* is regressed on *log*_10_(*conc*.) and *E* is calculated from the slope of this regression according to Eq. (6). If there is no data for a standard curve, *E* is estimated using the free *E* mode of Eq. (3) and averaged per amplicon.* The next step* is calculating *f*_0_ using the fixed *E* mode of Eq. (3) utilizing *E* calculated in the previous step. Followed by calculating *f*_0_% using Eq. (5)

In all modes, the initial cycle or cycles should be discarded if they deviate obviously from the baseline. Finally, *f*_0_% is calculated as a percentage of the predicted maximum fluorescence as shown in Eq. (5). A flowchart of the analysis process is shown in Fig. 2.

5 $$f_{0} {\% } = \frac{{f_{0} }}{{f_{m} }} \times 100$$

It is important to note that there are two sources of *E* that should be considered when using the fixed *E* mode:

1. In experiments lacking the data for a standard curve, *E* is calculated in the free *E* mode.
2. In experiments with the data for a standard curve, this data is converted to an approximate *f*_0_% using the fixed *E* mode (assuming *E* = 2). Then, the standard curve is built by regressing *log*_10_(*f*_0_%) on *log*_10_(*conc*.). Finally, *E* is calculated from the slope of the regression line using Eq. (6).
  6 $$E = 2^{1/Slope}$$

### Quantification

After calculating the C_T_, N_0_, Cy_0_, and* f*_0_% for all reactions in each of the 20 dilution curves, the C_T_, N_0_, Cy_0_, and* f*_0_% values were converted to predicted concentrations. For all methods, the predicted concentrations were calculated twice. Once considering the presence of a standard curve and once assuming the absence of a standard curve. This strategy was adopted to assess the performance of the used methods for experiments that either include or lack a standard curve, respectively.

#### Condition 1: considering the presence of a standard curve

The standard curve is built by regressing C_T_, *log*_10_(N_0_), Cy_0_, or *log*_10_(*f*_0_%) on *log*_10_(*conc*.). In the case of using C_T_ or Cy_0_, the slope of the regression line is negative because both C_T_ and Cy_0_ proportionate inversely with the concentration. While using *log*_10_(N_0_) or *log*_10_(*f*_0_%) produces a positive slope because N_0_ and *f*_0_% are directly proportional to the concentration. Finally, to retrieve concentrations, the regression equation is solved for *log*_10_(*conc*.) using the C_T_, *log*_10_(N_0_*)*, Cy_0_,* or log*_10_(*f*_0_%*)* values. And the obtained *log*_10_(*conc*.) is raised to the power of 10.

#### Condition 2: assuming the absence of a standard curve

Normally in this condition, a relative quantification is performed rather than an absolute one. However, for the aim of our study, a predicted concentration should be calculated for each reaction to be used in the performance indicators (described later). To solve this problem, the concentrations were predicted relative to the highest concentration (1st level) in each dilution curve. So, the predicted concentrations have the same scale as the true concentrations.

For the C_T_ and Cy_0_ methods:

7 $${\Delta }({C_{T}}\;or\; {Cy_{0}}) _{\left( i \right)} = ({C_{T}}\;or\; {Cy_{0}}) _{\left( i \right)} - Mean\left( ({C_{T}}\;or\; {Cy_{0}}) _{\left( {1^{st}\;level} \right)} \right)$$

8 $$Predicted\;conc._{\left( i \right)} = 2^{{ - {\Delta }(C_{T}\;or\;Cy_{0}) _{\left( i \right)} }} \times true\;conc._{{\left( {1^{st}\;level} \right)}}$$

For the *f*_0_% and LinRegPCR methods:

9 $$Predicted\;conc._{\left( i \right)} = \frac{({f_{0} \% \;or\;{\text{N}}_{0}) _{\left( i \right)} }}{{ Mean\left( ({f_{0} \% \;or\; {\text{N}}_{0}) _{{\left( {1^{st}\;level} \right)}} } \right) }} \times true\;conc._{{\left( {1^{st}\;level} \right)}}$$

### Performance indicators

The predicted concentrations calculated by the C_T_, LinRegPCR, Cy_0_, and *f*_0_% methods were compared with the true concentrations—concentrations obtained from the datasets—to evaluate the performance of these methods. Different performance indicators were needed to measure the performance of the tested methods from different aspects. Precision, which refers to the variation between replicates, was evaluated using the coefficient of variation and variance. Accuracy, which refers to the deviation of the predicted concentrations from the true concentrations, was assessed using the relative error and bias. The performance indicators were calculated as follows:

1. **Coefficient of variation (CV%).** CV%﻿ was calculated for each level of the dilution curves as follows [17]:
  10 $$CV\% = \frac{{SD\left( {predicted\;conc.} \right)}}{{Mean\left( {predicted\;conc.} \right)}} \times 100$$
2. **Variance.** Variance represents the within-level variance of the *log*_10_(*predicted concentration)* [7].
3. **Relative error (RE).** RE is the deviation of the predicted concentrations from the true concentrations [17].
  11 $$RE = \frac{predicted\;conc. -\; true\;conc.}{{true\;conc.}}$$
  The perfect value of RE is zero which indicates no error. While values greater or lesser than zero indicate error proportional to the absolute value. In this manner, if we take the average of RE for different reactions, negative values will negate the effect of positive values leading to a misleading average. Therefore, we calculated an absolute relative error that could be averaged.
  12 $$Absolute\;RE = \left| {Relative\; error} \right|$$
4. **Bias.** Bias is the ratio of the averaged predicted concentrations of the highest to the lowest levels [7]. For example, if the true concentration of the highest level in a given dilution curve is 10,000 and the true concentration of the lowest level is 10, then the perfect ratio for bias is 1000. Obviously, the perfect ratio varies between dilution curves according to the dilution rate and the number of levels. Therefore, a normalized bias was calculated by dividing the bias ratio by the perfect expected ratio of the respective dilution curve.
  13 $$Normalized\;Bias = \frac{{ Mean\left( {the \;highest\;predicted\;conc.} \right) }}{{Mean\left( {the \;lowest\;predicted\;conc.} \right)}} \div \frac{ the\;highest\;true\;conc.}{{the\;lowest\;true\;conc.}}$$
  Because the normalized bias may be greater or lesser than one—the optimum value—﻿an averaged normalized bias would be misleading as described earlier for relative error. So, we calculated an absolute bias that can be averaged with one indicating no bias and values greater than one indicating bias.
  14 $$Absolute\;Bias = e^{{\left| {\ln \left( {Normalized\;Bias} \right)} \right|}}$$
  For each of the previous performance indicators, a fold reduction was calculated to quantify the effect of using* f*_0_% instead of C_T_, LinRegPCR, or Cy_0_. Then, the geometric mean of the fold reduction was reported.
  15 $$Fold\;Reduction_{{\left( {PIx} \right)}} = \frac{{C_{T} ,\;LinRegPCR, \;or\; Cy_{{0 \left( {PIx} \right)}} }}{{f_{0} \%_{{ \left( {PIx} \right)}} }}$$
  where *PIx* is one of the performance indicators: CV%, variance, absolute RE, and absolute bias.

### Statistical analysis

Friedman test was performed to check if the distribution of each performance indicator—grouped by method, paired by dilution curve—shows a statistically significant difference. A significant Friedman test was followed by pairwise Wilcoxon signed-rank tests to identify the differences between methods. Then, *p* values were adjusted for alpha inflation using the Bonferroni correction. All statistical analysis was conducted using RStudio-2023.06.1-524 [23] and R programming language v4.3.1 [24]. A statistically significant difference was considered when (*p* value < 0.05). All R scripts containing the statistical analysis were provided in the Additional file 4.

## Results

The variation in the used qPCR datasets was intended to represent different templates, primers, master mixes, DNA-binding dyes, and qPCR instruments. Furthermore, the analysis process considered the varying objectives of the qPCR experiments from absolute to relative quantification, which influences the need for a standard curve. Finally, different performance indicators were used to measure the accuracy of the compared methods from different perspectives.

### Performance evaluation considering the presence of a standard curve

Upon comparing the methods considering experiments using a standard curve, it was clear that the *f*_0_% method offers more advantages than the C_T_ and LinRegPCR methods. Calculating the *f*_0_% reduced the variation between technical replicates indicating increased precision. This was evident by reducing the CV% of the C_T_ and LinRegPCR methods by 1.66 folds (*p* value < 0.001) and 1.65 folds (*p* value < 0.01), respectively. Moreover, the *f*_0_% reduced the variance of the C_T_ and LinRegPCR methods by 2.78 folds (*p* value < 0.001) and 2.61 folds (*p* value < 0.01), respectively. On the other hand, there was no statistically significant difference between the *f*_0_% and the Cy_0_ methods in both parameters. Furthermore, the C_T_, LinRegPCR, and Cy_0_ methods also didn't show a statistically significant difference between each pair of them regarding the CV% and variance.

**Fig. 3:**
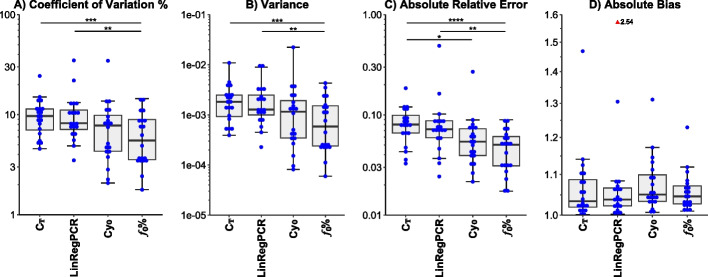
Performance evaluation considering the presence of a standard curve. **A** Coefficient of variation %: level-specific standard deviation as a percentage of the level-specific average. **B** Variance: within-level variance of the *log*_10_(*predicted concentration)*. **C** Absolute relative error: the absolute value of the deviation of the predicted concentrations from the true concentrations. **D** Absolute bias: the absolute value of the ratio of the average of the highest to the lowest predicted concentrations divided by the ratio of the highest to the lowest true concentrations. In all indicators, lower is better. *p value < 0.05, **p value < 0.01, ***p value < 0.001, and ****p value < 0.0001

Regarding accuracy, both *f*_0_% and Cy_0_ methods reduced the absolute relative error in comparison to the C_T_ method by 1.8 folds (*p* value < 0.0001) and 1.19 folds (*p* value = 0.022), respectively. Furthermore, only the *f*_0_% method decreased the absolute relative error regarding the LinRegPCR method by 1.71 folds (*p* value < 0.01). Regarding the other parameter of accuracy—absolute bias, the Friedman test was insignificant. Figure 3 outlines these results while Additional file 3: Table S1 shows the detailed performance of all methods on each dilution curve.

### Performance evaluation assuming the absence of a standard curve

In experiments lacking a standard curve, the *f*_0_% method showed potential advantage when compared to other methods specifically in the precision parameters. The *f*_0_% method increased the uniformity of replicates by lowering the CV% by 1.25 folds (*p* value < 0.01) against the Cy_0_ method, by 1.55 folds (*p* value < 0.01) against the LinRegPCR method, and by 1.76 folds (*p* value < 0.0001) against the C_T_ method. Moreover, the* f*_0_% method diminished the variance by 1.57 folds (*p* value < 0.001) regarding the Cy_0_ method, by 2.31 folds (*p* value = 0.033) regarding the LinRegPCR method, and by 3.13 folds (*p* value < 0.0001) regarding the C_T_ method.

**Fig. 4:**
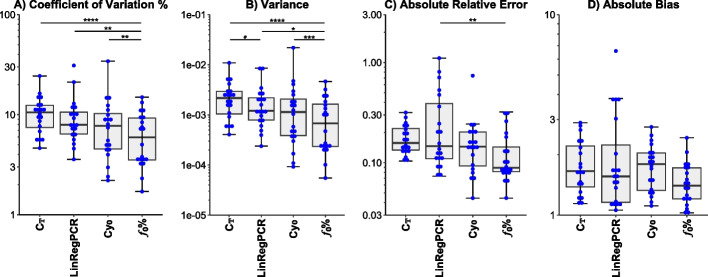
Performance evaluation assuming the absence of a standard curve. **A** Coefficient of variation %: level-specific standard deviation as a percentage of the level-specific average. **B** Variance: within-level variance of the *log*_10_(*predicted concentration)*. **C** Absolute relative error: the absolute value of the deviation of the predicted concentrations from the true concentrations. **D** Absolute bias: the absolute value of the ratio of the average of the highest to the lowest predicted concentrations divided by the ratio of the highest to the lowest true concentrations. In all indicators, lower is better. ^#^*p* value = 0.05, *p value < 0.05, **p value < 0.01, ***p value < 0.001, and ****p value < 0.0001

Regarding the accuracy related parameters, statistically significant differences were scarce. However, the *f*_0_% method minimized the absolute relative error in comparison to the LinRegPCR method by 1.83 folds (*p* value < 0.01). These results are briefed in Fig. 4 while the performance on each dilution curve is provided in Additional file 3: Table S2.

### Effect of the inflection cycle (***C***_***i***_) position on the performance of the ***f***_0_% method.

**Fig. 5:**
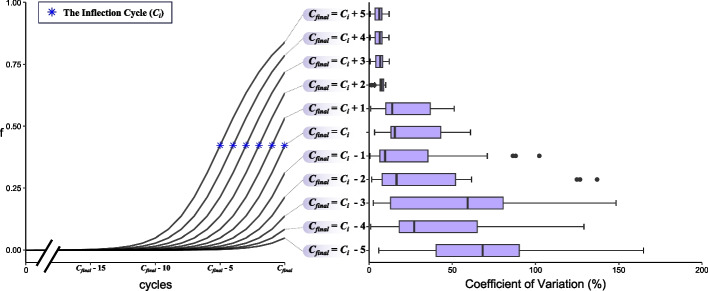
Effect of the position of the inflection cycle (*C*_*i*_) on the coefficient of variation (%). Reactions of the first levels of the 20 dilution curves were used to calculate *f*_0_% 11 times. Each time the reaction was trimmed at a different cycle relative to the *C*_*i*_ so that the final cycle (*C*_*final*_) ranged from (*C*_*final*_ = *C*_*i*_ − 5) to (*C*_*final*_ = *C*_*i*_ + 5). On the left side, amplification curves with different *C*_*i*_ ranging from (*C*_*final*_ = *C*_*i*_ − 5) to (*C*_*final*_ = *C*_*i*_ + 5). On the right side, a horizontal box plot representing the coefficient of variations (%) of the 20 dilution curves grouped by *C*_*i*_

As described earlier, all available cycle readings are used to obtain the *f*_0_% in the Fixed *E* Mode, however, sometimes all available cycles are not enough to calculate a precise *f*_0_%. The performance of the *f*_0_% depends on the inflection cycle (*C*_*i*_) position. It was assumed that the earlier the *C*_*i*_, the more precise the *f*_0_%. To check this assumption, the first levels of the 20 dilution curves were used to evaluate the performance of the *f*_0_% (represented as CV%) relative to the position of the *C*_*i*_. For each reaction, 11 predictions were performed that differed in the final cycle (*C*_*final*_) to be used in the model ranging from (*C*_*final*_ = *C*_*i*_ − 5) to (*C*_*final*_ = *C*_*i*_ + 5). We found that our assumption is true: an earlier *C*_*i*_ is associated with a more precise *f*_0_%. Generally, *f*_0_% is considered precise only when the *C*_*final*_ is two cycles or more after the *C*_*i*_ (*C*_*final*_ ≥ *C*_*i*_ + 2). Figure 5 shows the relationship between the performance of the *f*_0_% and the position of the *C*_*i*_ relative to the *C*_*final*_.

## Discussion

qPCR is a widely used technique to quantify minute amounts of specific nucleic acid. It allowed unprecedented advances in gene expression analysis and quantifying infectious pathogens [2]. The traditional method used to report the quantitative endpoint for that technique is the C_T_ method [5] which ignores most of the reaction data. To overcome the drawbacks of the C_T_ method, we introduce a new qPCR analysis method (*f*_0_%). The new method proved to be statistically more robust in most situations than the other compared methods. The *f*_0_% method outperformed the widely used C_T_ method in experiments that either contain or lack a standard curve. In experiments with a standard curve, the *f*_0_% reduced CV%, variance, and absolute relative error by 1.66, 2.78, and 1.8 folds, respectively. Moreover, when analyzing the experiments without using the standard curve data, the *f*_0_% reduced CV% and variance by 1.76, 3.13 folds, respectively. Finally, the *f*_0_% method was implemented in a macro-enabled Excel file available at https://github.com/Mahmoud0Gamal/F0-perc/releases with a user manual to describe how to professionally use the *f*_0_% method.

### Method development

The amplification curve of the qPCR has a characteristic sigmoid nature. Thereafter, many qPCR analysis methods adopted the sigmoid functions [12–14]. In 2004, Rutledge introduced the sigmoid function as a tool to analyze the qPCR curve. He used a four-parameter sigmoid equation with a symmetric nature that struggled to fit the asymmetric sigmoid curve of the PCR. To overcome this limitation, he excluded the plateau phase from the fit by choosing a cut-off cycle [12]. Later on, Spiess et al. introduced a flexible five-parameter sigmoid equation containing an asymmetry parameter. Spiess' equation increased the accuracy of the fit, but it retained a parameter responsible for the slope [13]. This slope parameter enables the calculation of efficiency from single reaction data and inherently quantification using this reaction-specific efficiency [25]. Although it corrects for efficiency variation and intuitively seems to be advantageous, quantification based on reaction-specific efficiencies tends to dramatically increase the variation between replicates and diminishes the reliability of the quantification. On the other hand, averaging the efficiency per each target achieves greater robustness and reliability [8].

The *f*_0_% method also utilizes a sigmoid equation (Eq. 3) with minor but critical modifications. In our equation, we substituted the constant in the denominator (the natural base—*e*) of the previous sigmoid equations [12, 13] with a variable that directly represents the starting efficiency (*E*). Moreover, redundancy was reduced by removing the parameter responsible for the slope, since its effect was transferred to the *E* parameter. Also, we retained the flexibility of the model by including a parameter for asymmetry (*D*). Here, *D* represents the rate of efficiency decay, a high value of *D* indicates a rapid reduction in cycle-to-cycle efficiency, associated with increased deceleration of the amplification rate. In contrast to *D*, *E* is the starting efficiency and it's not related to changes in efficiency from cycle to cycle.

Using reaction-specific efficiency in quantification was reported to increase the variation between replicates [8]. After validating this report, we avoided the direct quantification using reaction-specific efficiencies by calculating and averaging efficiencies from all amplicon-specific reactions using the free *E* mode. Then, taking this averaged efficiency to the fixed *E* mode to calculate the *f*_0_%. This scenario provides an estimate for efficiency when no standard curve is present while preventing the drastic errors caused by using reaction-specific efficiencies.

For accurate analysis of the qPCR curve, the background noise should be properly subtracted by a process called baselining. Therefore, a linear part (*ax* + *b*) was added to (Eq. 3) to allow the *f*_0_% method to subtract the background noise during the analysis. This approach was adopted instead of baselining separately before analyzing the qPCR curve to avoid reduced precision as noted earlier [25]. Indeed, our method of baselining is fairly accurate even if the background noise is shifting up or down assuming a linear shift. However, some qPCR reactions may show a complex non-linear baseline. In these reactions removing the first few cycles leaves a nearly linear baseline which is suitable for the *f*_0_% analysis.

The use of the *f*_0_% method was extended to correct for variations in the maximum fluorescence or the reaction volume. It is assumed that the maximum fluorescence is similar in all reactions containing similar amounts of the same PCR mixture. However, due to volume variations and fluctuations in the signal output from the PCR instrument, maximum fluorescence is not identical. This artifact occurs between different wells in the PCR instrument, and it is exaggerated when ROX normalization is ignored [26]. To remove the effect of this artifact on quantification, the *f*_0_% method reports the predicted initial fluorescence (*f*_0_) as a percentage of the predicted maximum fluorescence (*f*_*m*_) using (Eq. 5). Therefore, apparent differences in the maximum fluorescence would not impair the accuracy of the quantification.

### Performance

The performance of the *f*_0_% method was compared with the C_T_, LinRegPCR, and Cy_0_ methods which constitute the best subset of Friedman test in analyzing qPCR data as reported earlier by two independent benchmarking studies [7, 16]. The methods were tested for precision and accuracy using different indicators regarding various qPCR platforms and experimental designs. The overall precision of the *f*_0_% method was statistically superior to the C_T_ and LinRegPCR methods evidenced by reduced CV% and variance regarding both types of quantification (absolute and relative). In addition, considering relative quantification the* f*_0_% method was more robust than the Cy_0_ method. The superiority of the *f*_0_% method in precision is attributed to many factors. First, the *f*_0_% method—unlike the C_T_ and LinRegPCR—takes benefit of all cycle readings recorded by the qPCR instrument like other curve-fitting methods e.g., Cy_0_. Consequently, the *f*_0_% method doesn't depend on an arbitrary threshold that its position may change the results [13–15, 17]. Moreover, unlike other sigmoidal models [12, 13], the *f*_0_% and LinRegPCR methods depends on averaged efficiency per amplicon rather than quantification directly using reaction-specific efficiencies [15]. Finally, the *f*_0_% method is the first—based on our information—to normalize the result to the predicted maximum fluorescence.

In terms of accuracy, absolute relative error (RE) and absolute bias were calculated. Regarding the absolute RE, the *f*_0_% method offered substantial improvement over the C_T_ and LinRegPCR methods in experiments containing a standard curve. Moreover, when considering experiments lacking a standard curve, the *f*_0_% method continued to outperform the LinRegPCR method. However, in both types of experiments, there was no statistical difference between the *f*_0_% and Cy_0_ methods. This might be attributed to using the whole data points in the analysis of the *f*_0_% and Cy_0_ [14] but not for the C_T_ and LinRegPCR methods [5, 15].

Regarding the other accuracy parameter—absolute bias, depending on a standard curve made all methods produce relatively unbiased results that were very close to one. This was predicted as quantification using a standard curve nearly eliminates bias [7]. However, in experiments lacking a standard curve, the values of absolute bias were greatly deviated. Although bias is caused—in theory—by misestimating efficiency [7], methods that correct efficiency didn't show statistically significant enhancements in absolute bias. To interpret these unexpected findings, we shall consider the effect of dilution errors on bias. As these errors make the actual bias ratio vary greatly from the perfect expected ratio [8]. Therefore, it will be obvious that the most accurate methods will produce a deviated bias in case of dilution errors. In our case, we used publicly available datasets, so we had no control over their quality, and we relied on the quality control parameters stated by their authors.

### Limitations

The *f*_0_% method predicts the shape of the amplification curve which has a sigmoid nature. Sigmoid curves are characterized by the presence of one inflection point that separates the upper and the lower parts of the curve. To precisely predict the shape of this curve, we should provide enough data around the inflection point or inflection cycle (*C*_*i*_) [27]. However, in very rare occasions amplification curves may show late amplification—especially those with reduced efficiency—and fail to reach the *C*_*i*_ before the final cycle (*C*_*final*_) leading to insufficient data around this critical point. Therefore, the precision of the calculated *f*_0_% will be reduced.

In practical situations, most amplification curves will pass the *C*_*i*_. However, in very rare occasions—genes with very low expression along with reduced amplification efficiency—the amplification curve may fail to reach the *C*_*i*_. If a researcher encounters this situation, the reaction efficiency shall be enhanced. If it is not possible, the number of cycles could be increased. However, increasing the number of cycles will increase the chance of amplifying non-specific products. Therefore, increasing the number of cycles should be done with caution in these rare occasions only if efficiency improvements fail. Moreover, a high-resolution melt curve analysis must be examined carefully to identify any non-specific products.

Another limitation related to data availability is using the datasets that depend on DNA-intercalating dyes only for assessing the performance of the *f*_0_% method. Although the widespread usage of TaqMan probes in signal detection in qPCR experiments, we didn't find enough publicly available datasets to validate the performance of the *f*_0_% method on them.

### Applications

The goal of qPCR experiments is either absolute or relative quantification [3]. Absolute quantification relies on the presence of a standard curve with known concentrations as described earlier. On the other side, relative quantification could be performed without using a standard curve, but it requires the presence of one or more reference genes. Relative quantification is well described using the C_T_ method [5] and here we will describe how to perform two modes of relative quantification using the *f*_0_% method.

#### Fold change

Fold change is the ratio between the concentration of the target gene and the geometric mean of the concentrations of the reference genes of the same sample. Fold change is useful in experiments lacking a control group and could be calculated using Eq. (16).

16 $$Fold\;change = \frac{{f_{0} \%_{{\left( {Target\;gene} \right)}} }}{{Geometric\;Mean\left( {f_{0} \%_{{\left( {Reference\;genes} \right)}} } \right)}}$$

#### Normalized fold change

Normalized fold change is the ratio between the fold change of a given sample and the mean of the fold changes of the control group samples. In this case, the mean of the normalized fold changes of the control group samples should equal one. Normalized fold change is calculated using Eq. (17).

17 $$Normalized\;fold\;change_{{\left( {Sample\;x} \right)}} = \frac{{Fold\;change_{{\left( {Sample\;x} \right)}} }}{{Geometric\;Mean\left( {Fold\;change_{{\left( {Control\;group\;Samples} \right)}} } \right)}}$$

## Conclusions

Although the widespread usage of the C_T_ method in analyzing qPCR data, it suffers from many drawbacks. To address these limitations, we introduced the *f*_0_% method which utilizes all the available cycle readings to give more reliable results. The new method is based on a flexible sigmoid model that was modified to avoid the instability of the previous sigmoidal models. Indeed, our method demonstrated more robust and accurate results when compared with the C_T_, LinRegPCR, and Cy_0_ methods using a compiled multi-platform dataset. However, the enhanced performance of the *f*_0_% method comes at the cost of requiring a well-developed amplification curve that has at least two cycles post-inflection. Moreover, we facilitated the usage of the *f*_0_% by implementing the method in a user-friendly macro-enabled Excel file which can be easily downloaded and used by researchers. Overall, the *f*_0_% method offers a more reliable and accurate approach to qPCR analysis, with the potential to improve the accuracy of quantitative measurements in a variety of applications using qRT-PCR.

### Supplementary Information

**Additional file 1: Fig. S1.** Graphical representation of Dilution curves (1–10).

**Additional file 2: Fig. S2.** Graphical representation of Dilution curves (11–20).

**Additional file 3: Table S1.** Results of the performance indicators of the Ct, LinRegPCR, Cy0, and f0% methods considering the presence of the standard curve. **Table S2.** Results of the performance indicators of the Ct, LinRegPCR, Cy0, and f0% methods assuming the absence of the standard curve.

**Additional file 4:** R scripts used in the analysis process.

## Data Availability

All data analyzed during this study are imported from the qpcR R package as shown in the R scripts in Additional file 4.
