# Supplementary figures and images for: Introducing the f0% method: a reliable and accurate approach for qPCR analysis
Source: BMC Bioinformatics. 2024 Jan 11;25:17. doi: 10.1186/s12859-024-05630-y (PMC10782791; doi:10.1186/s12859-024-05630-y)

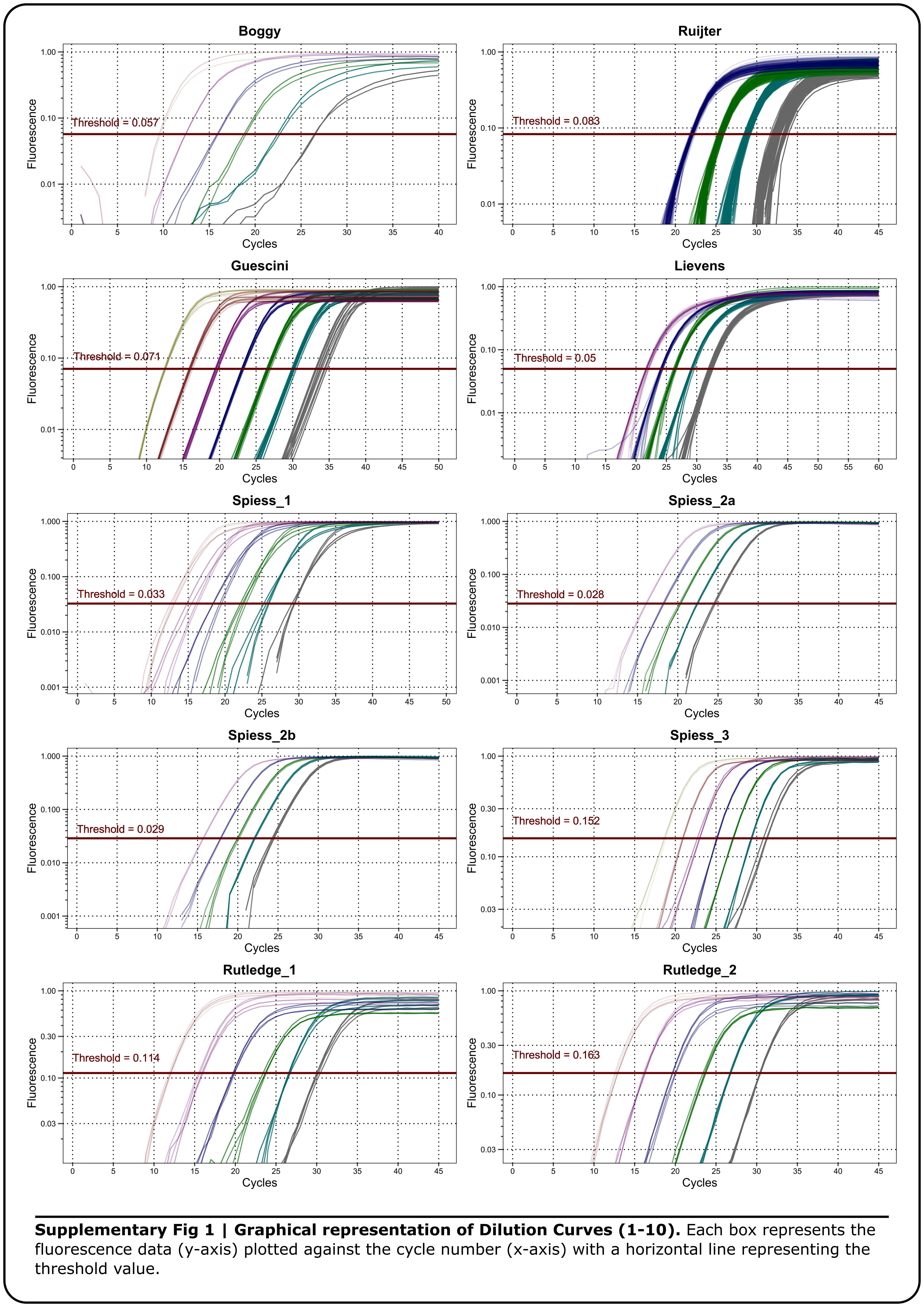

Supplement: Supplementary file 1 — Additional file 1: Fig. S1. Graphical representation of Dilution curves (1–10). [file 12859_2024_5630_MOESM1_ESM.png]

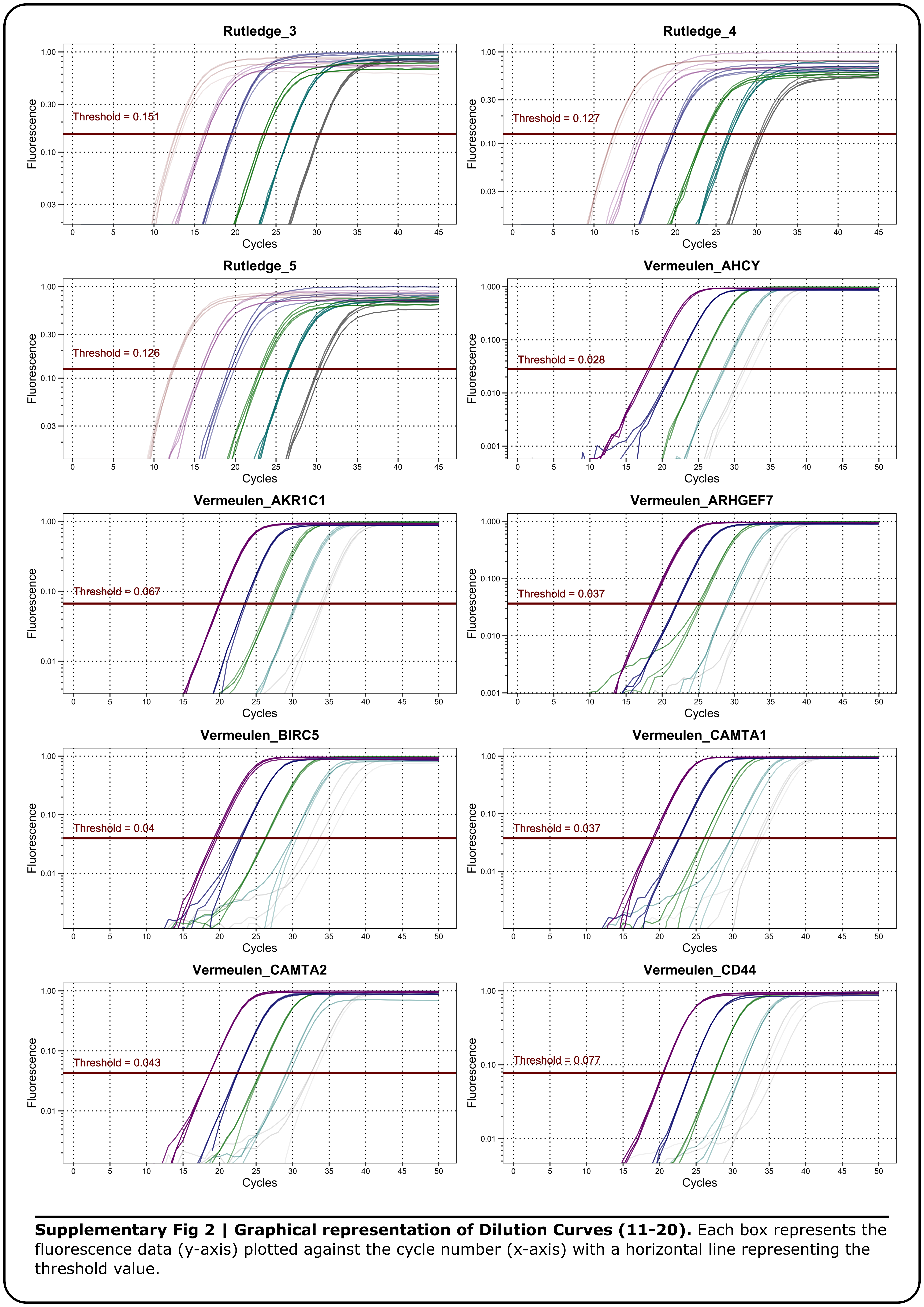

Supplement: Supplementary file 2 — Additional file 2: Fig. S2. Graphical representation of Dilution curves (11–20). [file 12859_2024_5630_MOESM2_ESM.png]
